# Predegenerating donor nerve for grafting using focused ultrasound neurotomy

**DOI:** 10.1038/s41598-025-00316-8

**Published:** 2025-05-04

**Authors:** Tak-Ho Chu, Nicolas Lasaleta, Siyun Li, Amanda McConnachie, Leonardo Molina, Saud Alzahrani, Laura Curiel, Samuel Pichardo, Rajiv Midha

**Affiliations:** 1 Department of Clinical Neurosciences, University of Calgary, Calgary, AB Canada; 2 Hotchkiss Brain Institute, University of Calgary, Calgary, AB Canada; 3 Department of Radiology, Cumming School of Medicine, University of Calgary, Calgary, AB Canada; 4 CSM optogenetics, Cumming School of Medicine, University of Calgary, Calgary, AB Canada; 5 Department of Biomedical Engineering, Schulich School of Engineering, University of Calgary, Calgary, AB Canada

**Keywords:** HIFU, Autograft, Predegeneration, Machine learning, Behavioural studies, Regeneration and repair in the nervous system, Peripheral neuropathies

## Abstract

Early experimental evidence suggests that predegenerated nerve graft enhance axonal growth compared to freshly harvested nerve graft in nerve gap repair. However, the practicality of surgically pre-conditioning nerves for grafting remains a major obstacle. Herein, we aim to investigate the feasibility of focal lesioning of nerve tissue using high-intensity focused ultrasound (HIFU) and compare the regenerative effects of predegenerated nerve grafts to freshly harvested grafts in a rat sciatic nerve transection and repair model. As a proof of principle, sciatic nerves were exposed for HIFU lesioning to increase targeting accuracy. HIFU induced focal thermal lesioning to the sciatic nerve, resulting in p75 and cJun up-regulation at the distal segment observed at 7 days post-sonication. All behavioural outcome metrics including von Frey sensory test and walking gait were similar between animals with freshly harvested and predegenerated nerve grafts except soleus muscle weight was significantly higher in the latter group. As well, similar axon histomorphometric results were found in 2-week survival animals. Our findings showed that it is possible to induce Wallerian degeneration in distal nerve for grafting using non-invasive HIFU. However, limited beneficial effects of predegenerated grafts were obtained compared to freshly harvested grafts in nerve gap repair.

**Supplementary Information:**

The online version contains supplementary material available at 10.1038/s41598-025-00316-8.

## Introduction

Nerve gap defects are commonly repaired with a freshly harvested donor nerve. However, some early experimental rodent studies show that transplantation of predegenerated nerve significantly improved axonal growth and motor reinnervation^1–3^. For example, after sciatic nerve gap injury and nerve grafting in Wistar rat, nerve pinch test showed that there is no delay for axons to regenerate into predegenerated nerve grafts compared to an estimated 3.6 day- delay in animals with freshly harvested grafts^3^. A follow-up study showed that even predegenerated acellular nerve graft enhances axonal growth into the graft, suggesting that predegeneration modifies the basal lamina or increases the availability of growth factors to regenerating axons, likely from deposition by previous resident cells during the degeneration process^4^. Indeed, recent studies showed that Schwann cells transited into a repair phenotype and are pro-regenerative one week after nerve injury, whereas their abilities gradually decline and are not supportive for axon regeneration at 10 weeks^5^. Repair Schwann cells activate cJun, a critical transcription factor for modulating the regeneration process^6^including up-regulation of brain- and glial- derived neurotrophic factors. Besides its trophic effect on regenerating axons, macrophage activation in the degenerated nerve also enhances angiogenesis which further supports the migration of Schwann cells from the transected ends into the graft^7^. It has been demonstrated that predegenerated nerve graft promotes migration of Schwann cells into the host nerve after transplantation^8^. These multifaceted benefits of predegenerated graft raise the question: why is a predegenerated nerve not being used for grafting in clinical practices? An obvious deterrent is the extra surgical procedure to injure a donor nerve days before the grafting surgery, which would add extra strain and cost to the health care system and increase patient risks for having open surgery. Additionally, contrasting experimental results showing a lack of beneficial effects or even inferior to fresh nerve graft^9–12^dampen the enthusiasm of using predegenerated grafts, partially due to incomplete analyses across all modalities, including axon count, electrophysiology, and functional studies^2^. However, the exact reason for these discrepancies remains unknown.

To circumvent the obstacle of invasive open surgery to create a predegenerated nerve graft, our first aim is to subject the nerve to high intensity focused ultrasound (HIFU) to determine whether thermal lesioning causes a focal and homogeneous neurotomy. HIFU has been applied to various disease treatment paradigms from tumour ablation to localized drug release^13^. It has been explored in clinic to manage a variety type of pain^14^. We have recently reported transient opening of blood nerve barrier using pulsed HIFU with systemic microbubble administration^15^, with supra-threshold sonication resulted in a significant structural damage to nerve immediately after treatment. A more simplistic approach is to use thermal energy induced by high energy of sonication; previous studies in a swine model demonstrated increasing energy settings using a magnetic resonance- guided focus ultrasound (MRgFUS) system caused graded lesion to nerves, as visualized by diffusion weighted imaging^16^. Nerve histology immediately post-ultrasound treatment also indicated clear focal endoneurial edema. Similarly, HIFU treatment in a rabbit nerve injury model suggested demyelination at 14 days after treatment, showing many vacant spaces in a cross section of the distal nerve^17^. Nevertheless, the suitability of using such post-sonicated distal nerve for grafting is unknown.

Recent developments of analytical tools for comparing axon histomorphometry using automated segmentation algorithms on entire nerve cross-sections and motion tracking algorithms to trace paw movements^18,19^ enable rapid and objective assessment of axon regeneration profile and functional recovery. Therefore, our second aim is to revisit the effects of HIFU-predegenerated nerve graft compared to fresh nerve graft in nerve gap repair using modern tools encompassing a complete analysis from axon histomorphometry to behavioural assessments.

## Results

### Successful thermal lesioning using high intensity focused ultrasound in Sprague-Dawley rats

We first determined the appropriate settings for neurotomy using HIFU at 1.5 MHz which is within the commonly used frequencies for therapeutic ultrasound applications^20^. Continuous HIFU exposure at 5 MPa or above pressure for 10 s consistently created a visible lesion on the nerve manifested by loss or “browning” of blood vessels whereas 3 MPa for 10 s did not cause any visible damage (Fig. 2A). Temperature monitoring revealed a peak change of 15–20 °C when 5 MPa was applied for 10 s (Fig. 2B). Immunostaining of the sonicated nerve segment revealed a homogenous up-regulation of p75 and cJun, and neurofilament segmentation distal to sonication site (arrowhead, Fig. 2C) at 7 days after sonication, suggesting successful neurotomy and predegeneration.

**Fig. 1:**
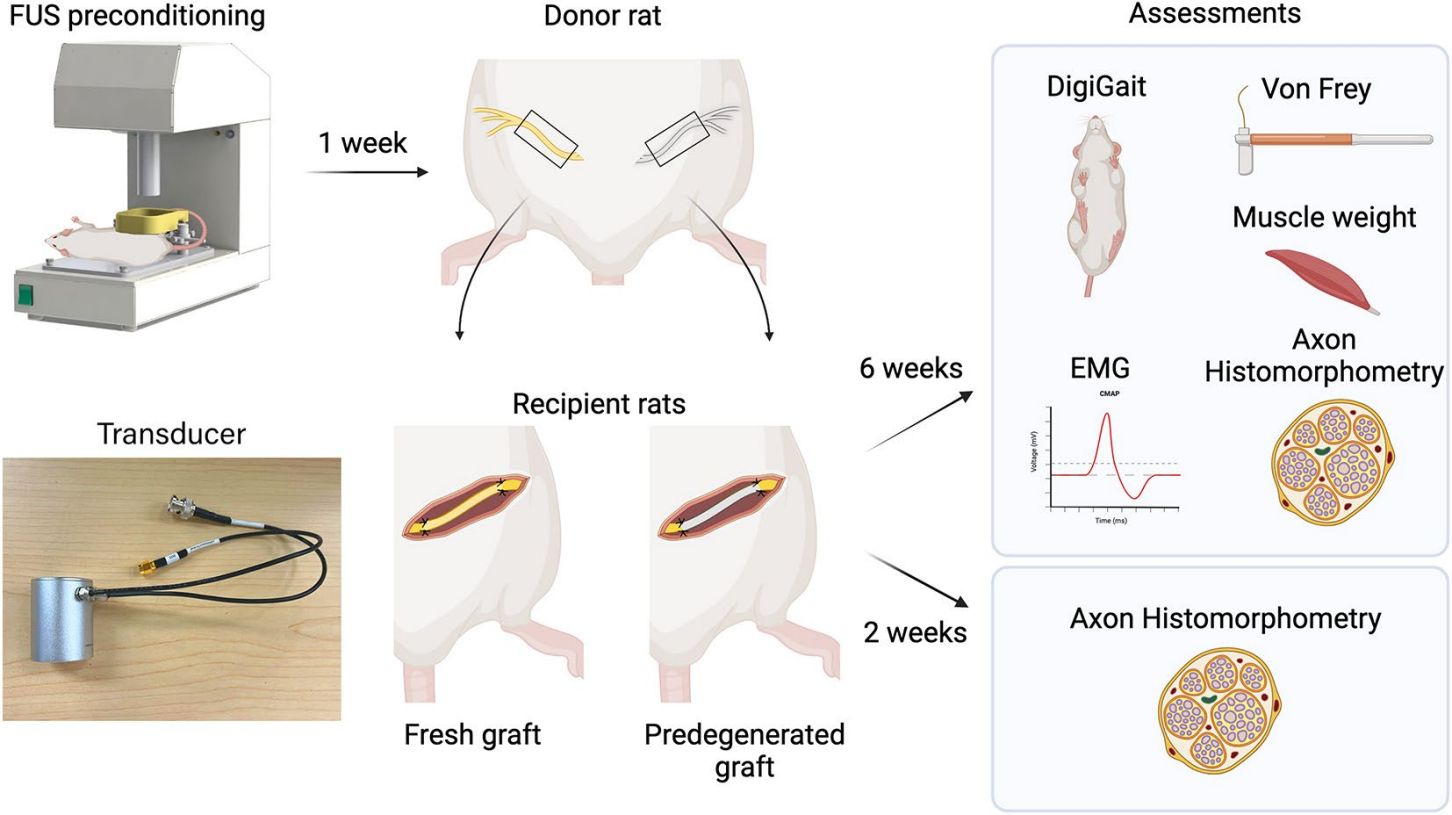
Schematic showing experimental approach. Nerve conditioning was achieved by bench top focused ultrasound system (transducer shown on left bottom). Schematics were created with BioRender.com.

**Fig. 2:**
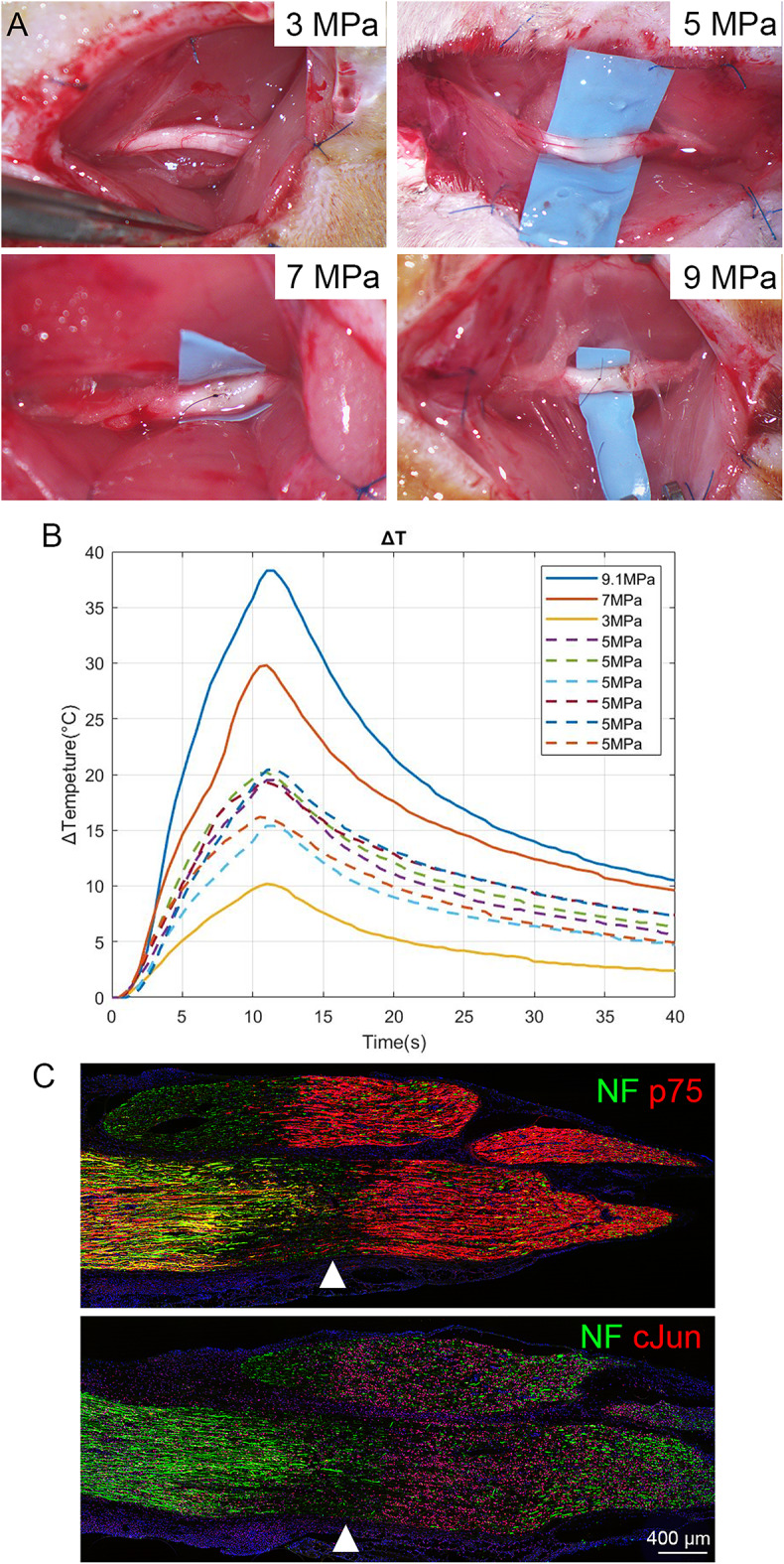
HIFU induced thermal injury and Schwann cell activation. Local lesion is shown in ascending pressure of HIFU settings, lesioning is noticeable at 5 MPA or above (**A**). Temperature change at 5 MPa, 1.5 Mhz for 10 s resulted in 15–20 °C rise locally (**B**). Immunostaining of nerve segment after HIFU shows up-regulation of p75 and cJun, indicating activation of Schwann cells at the distal segment right of the HIFU point on lesion (arrowhead, **C**).

### Similar functional and histomorphometric outcomes between fresh and predegenerated grafts six weeks after transplantation into recipient Lewis rats

**Fig. 3:**
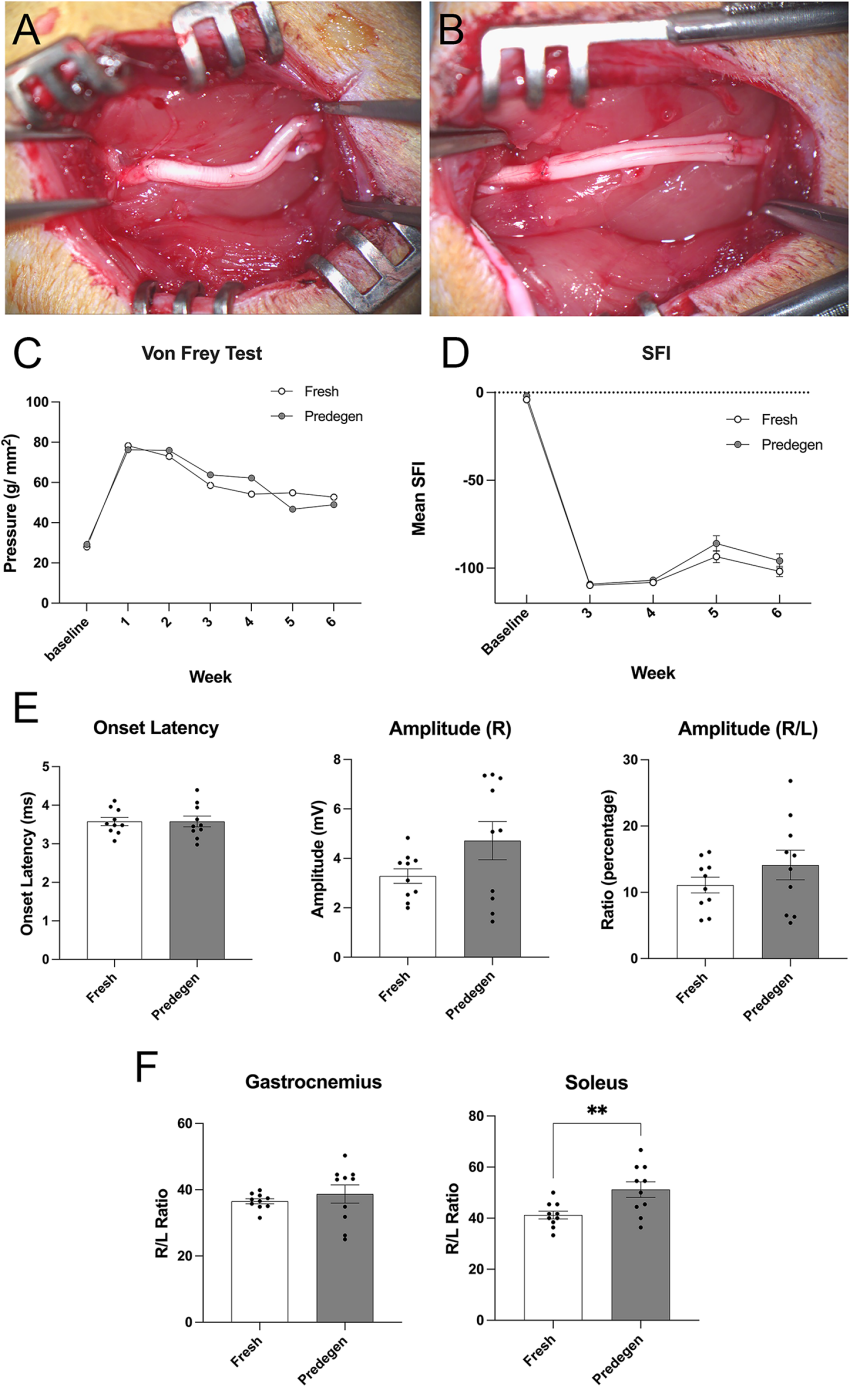
Similar functional outcomes after transplantation of fresh and predegenerated nerve grafts. Isogeneic transplantation of fresh (**A**) and predegenerated nerve grafts (**B**) into recipient Lewis rats. Functional assessments including von Frey test (**C**) and gait walking (**D**) show similar recovery profile. Electromyogram shows no significant difference in onset latency and peak amplitude between fresh and predegenerated grafts (**E**). Wet soleus muscle weight is significantly higher in animals with predegenerated grafts compared to that with fresh grafts but not in gastrocnemius muscle (**F**). SFI = sciatic functional index, R = right (ipsilateral side), L = left (contralateral side).

We then used a group of donor Lewis rats (*n* = 10) for supplying each 10 mm in length fresh (untreated) and 7 day- predegenerated nerves (sonicated at 5 MPa for 10 s) and grafted them into recipient Lewis rats after transecting and removing a 5 mm segment of host sciatic nerve (Fig. 3A & B). The Lewis rat is an inbred strain with similar genetic backgrounds, making it a suitable model for isograft transplantation studies^21^. Histological examinations of a short segment of each predegenerated donor nerve collected before transplantation showed homogenous p75 expression (Supp Fig. 2) and similar profile of cJun activation in Schwann cells, myelin clearance, macrophage infiltration, and laminin deposition distal to the sonication, similar to those in Sprague-Dawley using the same sonication parameters (Supp Fig. 3). Subsequent weekly assessments of von Frey test revealed both treatment groups followed a similar pattern with a loss of sensation in the first week following injury, and a gradual, incomplete sensory recovery over the 6-week post-injury period. Repeated two-way ANOVA showed no significant difference at any time points between the two groups (Fig. 3C). SFI calculations from walking gait analysis also revealed similar trend in regaining minimal recovery, with no statistical difference at any time points examined (repeated two-way ANOVA, Fig. 3D).

At the 6-week experimental endpoint, animals were subjected to electrophysiological recordings, and no difference in onset latency or amplitude (ipsilateral only or ipsilateral/contralateral ratio) between both treatment groups was noted (Fig. 3E). After euthanasia, gastrocnemius and soleus muscles were harvested and weighted. Analysis of the muscle weight ratio of the injured leg over the uninjured leg showed that the gastrocnemius in the animals with fresh and predegenerated grafts were similar, but the soleus regained more weight in the animals with predegenerated grafts compared to those with fresh graft (51.2 ± 3.06% vs. 41.24 ± 1.53%, *p* < 0.01, *t*-test, Fig. 3F).

**Fig. 4:**
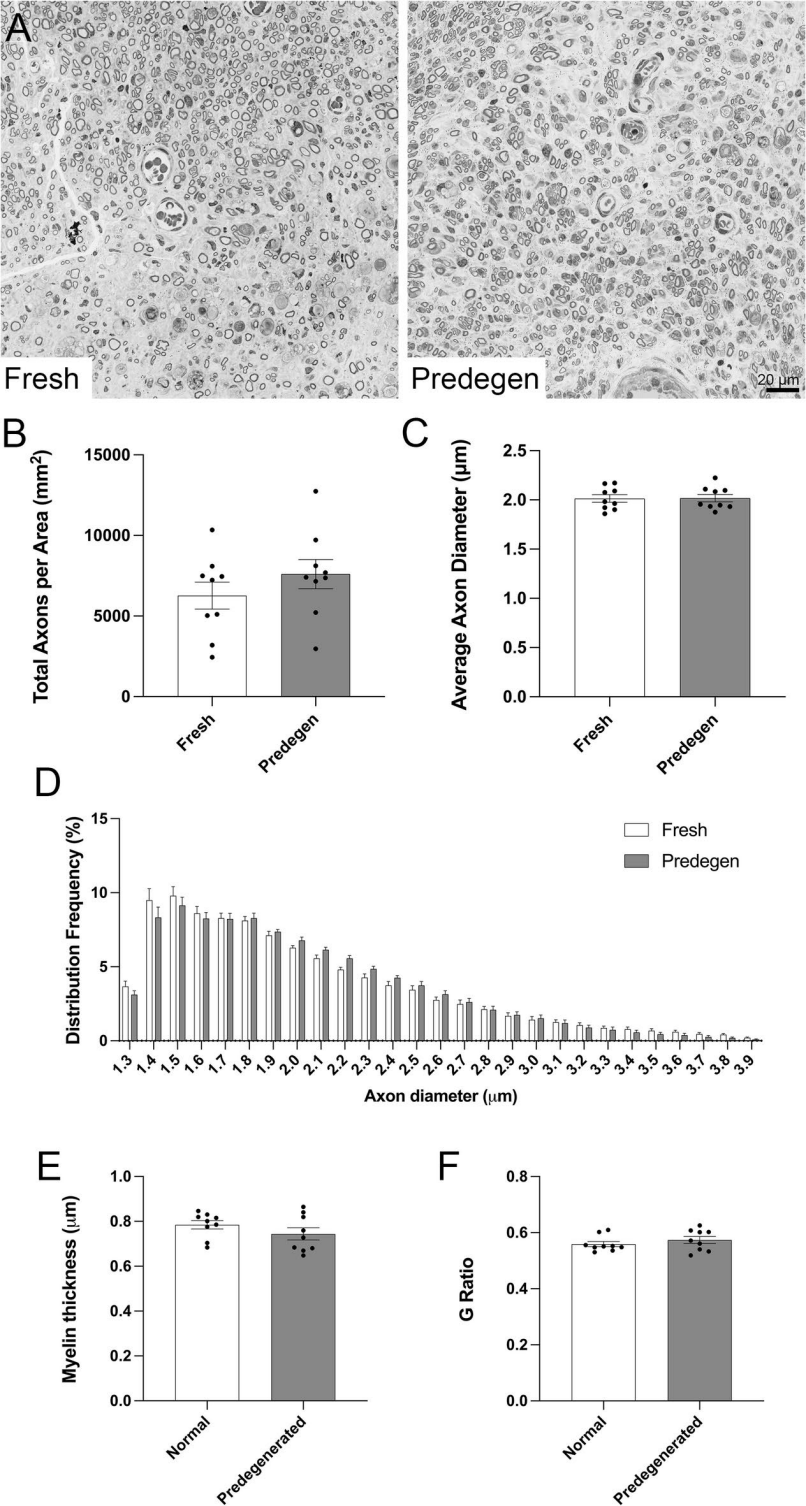
Axon histomorphometry from distal nerve segments in 6-week post-transplanted rats shows similar metrics between fresh and predegenerated nerve grafts. Representative semithin sections show numerous myelinated fibres in both groups (**A**). Total axon count (**B**), average axon diameter (**C**), distribution frequency (**D**), myelin thickness (**E**), and g-ratio (**F**) are all similar between fresh and predegenerated nerve grafts.

The nerve grafts were also harvested for histology, immunostaining with neurofilament showed axonal growth throughout the entire graft and into the distal host nerve (Supp Fig. 4). A segment of host nerve 2–3 mm distal to the nerve graft was collected and processed for histomorphometric analyses (Fig. 4A). Both groups showed similar morphometric features including total axons per area (Fig. 4B), axon diameter (Fig. 4C and D), myelin thickness (Fig. 4E) and g-ratio (Fig. 4F) between animals with fresh and predegenerated grafts (no significance using *t*-test).

### Similar histomorphometric outcomes between fresh and predegenerated grafts two weeks after transplantation into recipient rats

**Fig. 5:**
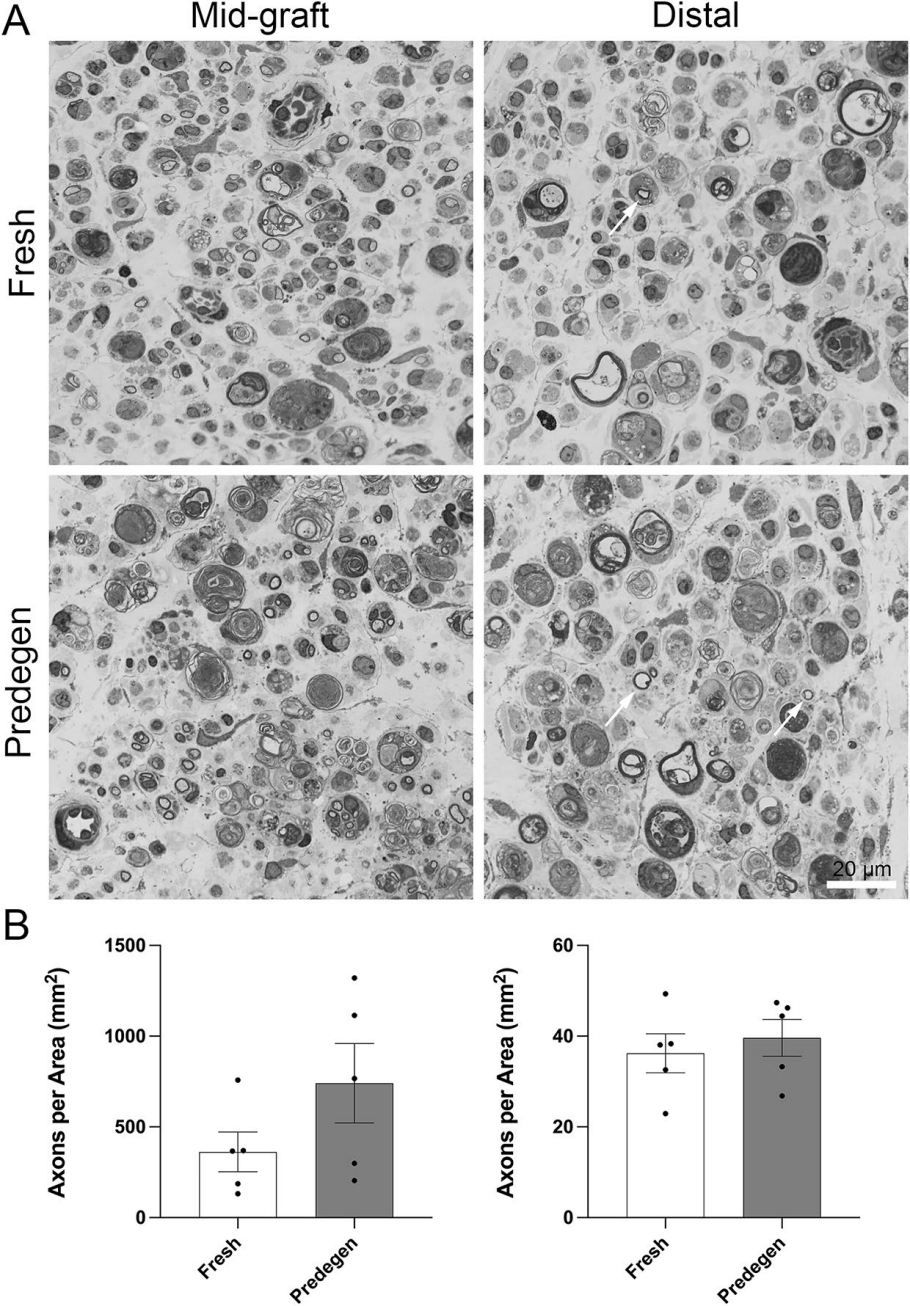
Axon histomorphometry from mid graft and distal nerve segments in 2-week post-transplanted rats shows similar number of axons between fresh and predegenerated nerve grafts. Representative semithin sections show few myelinated fibres in both groups, particularly in distal segments (arrows pointing to myelinated fibres, A). B. Manual counting of myelinated axons in mid-graft (left) shows higher number of counts in animals with predegenerated graft but not reaching statistical significance, whereas the number of myelinated axons is similar in distal segment (right).

Another group of animals (*n*= 5 in each group) was used to examine differences at two-week time point when axons should have regenerated 10 mm into transected and end-to-end repaired nerve^22^. A mid-graft segment and a host segment 2–3 mm distal to the coaptation were collected from animals two weeks after transplantation and used for histomorphometric analyses. Due to the presence of circular myelin debris at this time point, myelinated fibres were manually counted by a blinded observer instead of automated segmentation algorithm (Fig. 5A). The average number of axons per area in predegenerated mid-graft was higher but not statistically significant from fresh mid-graft (741.1 ± 218.9 vs. 362.3 ± 109.7, *p* > 0.05), probably due to large variations between samples. Only about one-tenth of axons were able to regenerate into the distal host nerve and average number of axons per area was comparable between the two groups (Fig. 5B); immunostaining with neurofilament showed similar results (Supp Fig. 5).

## Discussion

The present study addresses the repair of peripheral nerve gap injuries, where the nerve ends are too distant to reconnect effectively. In such cases, a donor nerve is used as a graft and is among the most effective technique to bridge a nerve gap and promote axonal regeneration^23^. Research into nerve gap repair exploring the regenerative effects of predegenerating nerve grafts began with the work of Ramon Cajal^24^. Many of the works that followed, however, show mixed results. Our studies revisited the notion using whole nerve cross section histomorphometric analyses assessing myelinated fibre counts, axon diameter, and myelin thickness. We also analyzed walking gait using machine learning for pose estimation, eliminating subjective error and reducing time for analysis^25^, in addition to muscle weight measurements, electromyography and sensory function tests.

The enhanced regenerative ability of predegenerated nerve graft is primarily attributed to Wallerian degeneration and Schwann cell activation, both of which create a favorable environment for regeneration. Wallerian degeneration occurs in the distal stump following nerve axotomy, initiating a cascade of cellular responses that promote axon regeneration. During this process, axons are fragmented, myelin disintegrates, macrophages infiltrate locally, and Schwann cells adopt a repair phenotype. Additionally, various neurotrophic and transcription factors are upregulated to respond to the injury, fostering a pro-regenerative environment. This environment facilitates axonal regeneration by allowing regenerating axons to elongate along Bungner Bands to reach their target endpoints^26^. Therefore, studies have shown that grafting with a nerve predegenerated for 1–3 weeks promoted faster recovery^3,27^, despite no different from fresh graft at longer survival period^27,28^. Specifically, Bertelli et al. and Kerns et al. reported 6 days delay in finger flexion recovery and 3 days delay in nerve pinch test in animals with fresh grafts, respectively^3,29^. While other studies reported a higher number of axon count in distal nerve repaired with predegenerated graft compared to fresh graft^2,30^, predegenerated graft was also shown to preferentially influence muscle reinnervation compared to fresh graft using retrograde labeling of motor neurons^1^. The authors suggested that myelin clearance, laminin deposition, and growth factors release in 2 week- predegenerated nerve grafts provide a cumulative environment to interact with regenerating axons.

Conversely, other experimental studies have reported no significant improvement in axonal regeneration with predegenerated nerve grafts compared to fresh nerve grafts^9–12^with the exception when recipient nerve was also injured prior to repair with predegenerated grafts^2,9^, likely due to preconditioning of regenerative responses in neurons^31^. Our overall results also demonstrated that the effects of a predegenerated graft is not significantly different from a fresh graft six weeks after transplantation except for a significant increase in soleus muscle weight in animals receiving predegenerated grafts (Fig. 3F). Whether predegenerated grafts might improve motor axon reinnervation of muscle warrants further investigation. The exact mechanism for the discrepancy is not known, Dubuisson et al. suggested that the superior regenerative ability of rodent axons may have masked the true beneficial effects of predegenerated graft^10^. In fact, our two-week time point experiment showed a higher number of myelinated fibres in the mid portion of predegenerated graft despite statistically not significant, indicating that predegeneration may have influenced the onset of fibre crossing into the graft but not the overall speed of regeneration, as shown by the similar number of myelinated fibres in the distal part of host nerves in the two-week samples (Fig. 5). The only study that compared fresh graft with predegenerated graft in non-rodent model was conducted in rabbit using facial nerve repair^30^which demonstrated a higher number of axon count in 3-week predegenerated nerve graft compared to non-degenerated graft. However, rate of regeneration in rabbit is comparable to that in rodents which is about 3–4 mm per day^32,33^. A large animal model, such as a sheep or swine model^34,35^, which grows at a comparable rate to humans at 1 mm/day, should be considered.

HIFU has been used in neural research and surgeries, including peripheral nerve ablation and nerve conduction block^36,37^. As a proof of principle, we exposed the nerve for precise targeting due to the small size of a rat sciatic nerve and to avoid potential skin lesion^38^. We showed segmentation of axons distal to the sonicated site and up-regulation of cJun and p75 at one week after treatment (Fig. 2C). A recent study by Rodríguez-Meana et al. also demonstrated functional impairment and structural damage after applying low frequency HIFU with pressures below 5 MPa on exposed rat sciatic nerve with a total accumulated treatment time of 25 s^38^. However, Walker et al. achieved accurate targeting using transcutaneous MRgFUS for navigation and lesioning^16^. They demonstrated significant changes in diffusion weighted imaging on piglets’ sciatic nerves sonicated by a 1.2 MHz transducer for 20 and 27 s. Similarly, repetitive treatment with transcutaneous low-intensity focused ultrasound has been demonstrated to improve functional recovery after a 10-mm autograft repair^39^. Due to the absence of significant improvement in predegenerated nerve graft, we did not further investigate transcutaneous sonication and how it affects tissue homeostasis around the treatment site. It is also unknown how HIFU affects pain; however, we did not observe abnormal behaviour suggestive of pain in our donor Lewis rats. Other HIFU non-thermal ablative methods, such as histotripsy^40^, seem not to be an adequate alternative to thermal-based HIFU lesioning. Histotripsy uses very short (few microseconds) and very intense (negative pressure between 26 and 30 MPa) ultrasound pulses to produce controllable cavitation activity capable of liquifying soft tissue. However, tissues with a high content of collagen, such as large nerves, are immune to the effects of histotripsy^40^, unless coupled with prolonged collagenase pretreatment and high heat^41,42^, making histotripsy more suitable for ablation of tissues softer than nerves in vivo.

In summary, we demonstrated that thermal ablation with HIFU at 1.5 MHz, 5 MPa for 10 s on exposed nerve induced focal and homogenous lesioning of donor nerve, as evidenced by up-regulation of cJun and p75 one week after sonication. However, nerve gap repair with a predegenerated nerve graft did not produce a significant improvement over a fresh nerve graft. We conclude that the other strategies that can promote regeneration across nerve gap should be pursued.

## Materials and methods

### Ethical statement

All animal experiments were approved by the University of Calgary Animal Care Committee (AC20-0172). We confirm that all methods were performed in accordance with the relevant international, national and institutional guidelines and regulations. Additionally, we confirm that the study is reported in accordance with ARRIVE guidelines.

### Lesion model

To induce nerve predegeneration in rat model, adult Sprague-Dawley rats (male or female, 225–300 g, Charles River, Canada) were anesthetized using 2% isoflurane with oxygen in prone position. Analgesics buprenorphine (0.05 mg/kg, sc) and Meloxicam (1 mg/kg, sc) were given before surgery, Meloxicam (1 mg/kg) was given subcutaneously for 2 days after surgery. Under aseptic conditions, the mid-thigh area was shaved and disinfected with 70% isopropanol followed by betadine. The right sciatic nerve was isolated and raised with a piece of latex cut from examination glove and placed underneath the nerve. Rats were placed under a bench-top, stereotactic-guided ultrasound platform (RK50, FUS Instruments, Canada). Sciatic nerve was then covered with sterile ultrasound gel (Lubricating Jelly, Medline, USA) to ensure coupling, and contact with the bottom of a polyimide membrane water chamber filled with deionized, degassed water, with a transducer (Fig. 1, FUS Instruments, Canada) immersed in the chamber. Continuous HIFU was delivered at the central frequency of the transducer, which works most efficiently at 1.5 MHz, at 3,5,7 and 9.1 MPa for 10 s. The transducer has a beam of dimensions of 0.98 mm (lateral) and 5.46 mm (axial) with a maximum cross-sectional area of 4.15 mm^2^at a normalized power at -6 dB as determined previously^43^. Acoustic pressures were pre-determined by positioning a 0.2 mm- calibrated needle hydrophone (Precision Acoustics, UK) at the focal point of the transducer in a water tank setup. The voltage recorded by the hydrophone was subsequently converted to pressure. Nerve was visually inspected to confirm success of lesioning. The local temperature change was monitored and collected by a fiber optic temperature sensor (Neoptix Reflex, Neoptix Inc, Canada) with a temperate probe (T2 Fiber Optic Temperature Probe, Neoptix Inc, Canada) placed in close proximity (~ 1–2 mm proximal) to the target site.

### Nerve graft

The experimental plan is outlined in Fig. 1. Inbred Lewis rats were used for nerve graft experiments to minimize allogeneic rejection. Donor animals were used to provide fresh graft and predegenerated graft from each animal. Adult female Lewis rats (225–250 g, Charles River, Canada) were used (*n* = 10). Sciatic nerve was predegenerated according to the lesion model using 1.5 MHz at 5 MPa for 10 s as described above. Animals were kept for 7 days before nerve harvest.

On day 7, donor rats were euthanized with overdosed sodium pentobarbital, 10 mm lengths of right sided predegenerated nerve (2 mm distal to neurotomy) and left sided naïve nerve were harvested to provide grafts for 2 recipient adult female Lewis rats. The proximal stump of the predegenerated nerves were harvested for histology to rule out the inclusion of local lesion zone. For recipient rats, only the right sciatic nerve was operated. Briefly, under deep anesthesia, 0.5 cm of sciatic nerve was cut and removed, donor nerve graft was then transplanted into the nerve gap and secured with multiple 10 − 0 sutures. Animals were kept for 2 weeks for histology or 6 weeks during which behavioural assessment were performed weekly as detailed below.

### Behavioural assessment

DigiGait walking analysis and nociceptive sensory assessment were performed weekly to monitor functional recovery as previously described^44^. Briefly, all assessments and analyses were blinded to the experimenter until the experiment was complete. All animals underwent training sessions and baseline recording before the surgery. For DigiGait imaging, the DigiGait (Mouse Specifics Inc., Boston, MA) imaging system was used to assess changes in sciatic nerve function before and after treatment. Video footages were captured when the animals were in continuous stride moving at 1.5 cm per second. The footages were then loaded into machine-learning tracking algorithm DeepLabCut (DLC)^19^to generate print length and toe spreads (Supp Fig. 1). To create the dataset for training, 10 animals were randomly selected and 14 distinct points of interests from their frames were labelled using the labelling tools of DLC: the tips of each digit on both hind paws when planted (10), the back of each hind paw print when planted (2), the base of the tail (1), and the chest (1). The network was then trained using the intrinsic data augmentation method “imaug” and the network architecture “resnet_101” for 200,000 iterations. When training was complete, the trained network was evaluated by calculating the likelihood of correct body part labeling (p) and using the root mean square error (RMSE) between the manually labeled markers and predicted markers of the algorithm. A high p-cutoff value at 0.9 and a low RMSE at 4.65 were used to ensure precise prediction. The generated dataset was further visually inspected to confirm accuracy of the tracking. To extract the data from the resulting H5-type files from DLC, we developed a python script to average print length and toe spread measurements above a specified confidence level (0.9) and store those values into a new file. Then, using the Bain-Mackinnon-Hunter formula^45^, we calculated sciatic functional index (SFI) from the resulting average measurements using a custom-script (Supplementary data or https://github.com/leomol/SFI).:

$$\:-37.2\:\left(\frac{EPL-NPL}{NPL}\right)+104.4\:\left(\frac{ETS-NTS}{NTS}\right)+45.6\:\left(\frac{EIT-NIT}{NIT}\right)-8.8$$

For nociceptive sensory assessment, the simplified up-down method (SUDO) using von frey filaments was adopted^46^. Briefly, in a quiet room, animals were placed in acrylic chambers on an elevated wire mesh grid and were left to acclimatize for 30 min before testing. Beginning with the tenth filament, the filament was applied to the lateral hind paw until the filament bent to elicit a withdrawal response. Then four more filaments were used depending on the response: if the animal withdrew at least 3 out of the 5 applications, this was evaluated as a positive response in which a smaller filament would be used next; and if the animal did not withdrawal 3 out of the 5 applications, this was evaluated as a negative response and a larger filament would be used next. This procedure was done on one animal, before repeating with the subsequent animals. Filament number was then converted to pressure by calibration of filaments as previously described^44^.

### In vivo electrophysiology

Electrophysiological assessments were performed at 6 weeks after injury, and compound muscle action potential (CMAP) was measured (Cadwell Laboratories, USA) as previously described^15^. Briefly, bipolar electrode was used to stimulate the proximal sciatic nerve at 2.5 mA (supramaximal stimulation) with 50 ms square wave pulse and evoked electromyogram activity was recorded from the lateral gastrocnemius muscle with filter cutoff at 10-10k Hz. Peak amplitude and onset latency was recorded and averaged from three independent recordings for each animal.

### Tissue processing

Animals were euthanized with lethal doses of sodium pentobarbital after electrophysiology recordings. Gastrocnemius and soleus muscles from both legs were dissected and weighted. The host nerves with nerve grafts were collected and fixed with 4% paraformaldehyde in 0.1 M phosphate buffer overnight at 4 °C and subsequently placed in 30% phosphate buffered sucrose until sectioning. After the samples had sunk, nerve samples were embedded in optimum cutting temperature compound (VWR, USA), frozen in pre-cooled isopentane, and cut in longitudinal sections at a thickness of 10 μm on a cryostat. The sections were collected on SuperFrosted Plus slides (VWR, USA) and stored at -20 °C for later use. For immunohistochemistry, sections were washed with 0.01 M phosphate buffered saline (PBS), blocked with 5% normal serum in diluent containing 0.3% Triton X-100 and 1% bovine serum albumin in 0.01 M PBS for 1 h at room temperature. Primary antibodies including neurofilament (NF200, 1:10000, #822601, Biolegend, USA), p75 (1:500, #839701, Biolegend, USA), cJun (1:200, #9165, Cell signaling Technology, USA), laminin (1:200, #sc-20777, Santa Cruz, USA), iba1 (1:200, #019-19741,Wako, Japan), and myelin protein zero (1:200, #ab31851, Abcam, USA) were diluted with diluent and incubated overnight at 4 °C. After three washes (15 min each) in PBS-tween 20 (0.05%, PBS-T), the sections were incubated in the corresponding fluorescent conjugated secondary antibodies (1:500, Invitrogen, USA) with 4′,6-diamidino-2-phenylindole (DAPI, 1:1000) in PBS for 1 h at room temperature. After 3 washes in PBS-T, sections were mounted with PermaFluor mounting medium (Thermo Scientific, USA) and imaged with a slide scanner (VS110, Olympus, Japan).

Additionally, for 6-week samples, a 2–3 mm nerve segment 2 mm distal to the distal coaptation was harvested and fixed in 2.5% glutaraldehyde overnight at 4 °C. For 2-week sample, an additional 2–3 mm mid-graft segment was also collected. The specimens were post-fixed in 2% osmium tetroxide followed by dehydration with graded acetone and then embedded in Epon resin. Then cross-sections were cut at 1 μm thickness and stained with 0.5% Toluidine Blue O. Images were analyzed using SEM prediction model in AxonDeepSeg according to the instructions^18^. Briefly, whole nerve semithin sections were scanned with a slide scanner (VS110, Olympus, Japan) at 100x. The scanned images were then processed in Adobe^®^ Photoshop^®^ to reduce image size by 50%, remove unwanted structures, convert to black and white, and invert colour. Then images were analyzed using the SEM prediction model in AxonDeepSeg, circle axon shape model, and an overlap value of 48 ^18^. Reducing image sizes by 50% reduced the processing time while maintaining accurate segmentation and a pixel ratio of 0.128 μm/pixel was used to conserve the scale at 100x. After segmentation, the Napari GUI tools were used to make any adjustments to the segmentation and the morphometric measurements were automatically determined and calculated. We also developed a python script that returns a filtered data set from the resulting CSV-type files based on user ranges for eccentricity, solidity, axon diameter, axon area, myelin thickness, and myelin area that were manually determined.

### Data analysis

Results are expressed as mean ± standard error of the mean (SEM). Quantitative data were analyzed using Prism 10 (GraphPad, USA). Repeated measure two-way analysis of variance and the Tukey multiple comparisons post-hoc test or Student’s t-test, where appropriate, were used to determine the statistical significance of differences among the means. A value of *p* < 0.05 was considered significant.

## Electronic supplementary material

Below is the link to the electronic supplementary material.

Supplementary Material 1

Supplementary Material 2

## Data Availability

The datasets generated during and/or analysed during the current study are available from the corresponding author on reasonable request.
