# Supplementary material for: Predegenerating donor nerve for grafting using focused ultrasound neurotomy
Source: Sci Rep. 2025 May 4;15:15581. doi: 10.1038/s41598-025-00316-8 (PMC12050296; doi:10.1038/s41598-025-00316-8)
Supplement: Supplementary file 1 — Supplementary Material 1 [file 41598_2025_316_MOESM1_ESM.docx]

# Batch calculation of the sciatic functional index (SFI) from DLC generated files with columns

# L/R * Thumb/Pinky/Index/Ring/Heel/Middle/Thumb/Pinky/Index/Ring/Heel/Middle * x/y/likelihood

# 2023-12-04. Leonardo Molina.

# 2023-12-18. Last modified.

# Script description:

# -Searches a project folder recursively for DLC generated h5-type files.

# -For each file found, creates a file at the same location (same name except DLC replaced by SFI) with columns SFI and ToeSpread/IntSpread/PrintLength for NormalLeft/InjuredRight or NormalRight/InjuredLeft.

# -Creates a file with the average of those measurements for each file, for frames where likelihood for all parts exceed a given threshold.

# Example installation using Anaconda/Miniconda:

# conda create -n 3.9 python=3.9

# conda activate 3.9

# conda install pytables

# python sfi.py

from pathlib import Path

import math

import numpy as np

import pandas as pd

def process(projectFolder, meansFilename, saveFullPath, framesOutputTypes, meansOutputTypes, confidence, injurySide):

# Search recursively for hdf5 files.

paths = list(Path(projectFolder).glob('**/*DLC*.h5'))

nPaths = len(paths)

# Process injury side.

injured, normal, I, N = (('Injured', 'Right'), ('Normal' , 'Left'), 'R', 'L') if injurySide == 'Right' else (('Injured', 'Left'), ('Normal' , 'Right'), 'L', 'R')

# Create headers.

framesMultiIndex = pd.MultiIndex.from_tuples(

[('Distance', target, condition, side) for target in ('ToeSpread', 'IntSpread', 'PrintLength') for condition, side in (normal, injured)] +

[('Proportion', target) for target in ('ToeSpread', 'IntSpread', 'PrintLength')] +

[('SFI',)]

)

# Dataframe for averages.

filesDf = pd.DataFrame(0, columns=framesMultiIndex, index=range(nPaths), dtype=np.float64)

# Reusable functions.

distance = lambda row, key1, key2 : math.sqrt(((row[key1, 'x'] - row[key2, 'x']) ** 2) + ((row[key1, 'y'] - row[key2, 'y']) ** 2))

proportion = lambda injured, normal : (injured - normal) / normal

# Process files found.

for pathId, path in enumerate(paths):

# Read.

df = pd.read_hdf(str(path))

df = df.droplevel('scorer', axis=1)

nFrames = len(df)

# Dataframe for frame calculations.

framesDf = pd.DataFrame(0, columns=framesMultiIndex, index=range(nFrames), dtype=np.float64)

# Distanes for each frame.

framesDf[('Distance' , 'ToeSpread' , *normal )] = df.apply(lambda row: distance(row, N + 'Thumb', N + 'Pinky' ), axis=1)

framesDf[('Distance' , 'IntSpread' , *normal )] = df.apply(lambda row: distance(row, N + 'Index', N + 'Ring' ), axis=1)

framesDf[('Distance' , 'PrintLength', *normal )] = df.apply(lambda row: distance(row, N + 'Heel' , N + 'Middle'), axis=1)

framesDf[('Distance' , 'ToeSpread' , *injured)] = df.apply(lambda row: distance(row, I + 'Thumb', I + 'Pinky' ), axis=1)

framesDf[('Distance' , 'IntSpread' , *injured)] = df.apply(lambda row: distance(row, I + 'Index', I + 'Ring' ), axis=1)

framesDf[('Distance' , 'PrintLength', *injured)] = df.apply(lambda row: distance(row, I + 'Heel' , I + 'Middle'), axis=1)

# Proporions for each frame.

framesDf[('Proportion', 'ToeSpread' )] = proportion(framesDf[('Distance', 'ToeSpread' , *injured)], framesDf[('Distance', 'ToeSpread' , *normal)])

framesDf[('Proportion', 'IntSpread' )] = proportion(framesDf[('Distance', 'IntSpread' , *injured)], framesDf[('Distance', 'IntSpread' , *normal)])

framesDf[('Proportion', 'PrintLength')] = proportion(framesDf[('Distance', 'PrintLength', *injured)], framesDf[('Distance', 'PrintLength', *normal)])

# SFI for each frame.

framesDf[('SFI')] = -38.3 * framesDf[('Proportion', 'PrintLength')] + 109.5 * framesDf[('Proportion', 'ToeSpread')] + 13.3 * framesDf[('Proportion', 'IntSpread')] - 8.8

# File average.

parts = ('LThumb', 'LPinky', 'LIndex', 'LRing', 'LHeel', 'LMiddle', 'RThumb', 'RPinky', 'RIndex', 'RRing', 'RHeel', 'RMiddle')

columns = [(part, 'likelihood') for part in parts]

inStance = df.loc[:, columns].min(axis=1) >= confidence

filesDf.loc[pathId, ('Distance' , 'ToeSpread' , *normal )] = framesDf.loc[inStance, ('Distance' , 'ToeSpread' , *normal )].mean(axis=None)

filesDf.loc[pathId, ('Distance' , 'IntSpread' , *normal )] = framesDf.loc[inStance, ('Distance' , 'IntSpread' , *normal )].mean(axis=None)

filesDf.loc[pathId, ('Distance' , 'PrintLength' , *normal )] = framesDf.loc[inStance, ('Distance' , 'PrintLength', *normal )].mean(axis=None)

filesDf.loc[pathId, ('Distance' , 'ToeSpread' , *injured)] = framesDf.loc[inStance, ('Distance' , 'ToeSpread' , *injured)].mean(axis=None)

filesDf.loc[pathId, ('Distance' , 'IntSpread' , *injured)] = framesDf.loc[inStance, ('Distance' , 'IntSpread' , *injured)].mean(axis=None)

filesDf.loc[pathId, ('Distance' , 'PrintLength' , *injured)] = framesDf.loc[inStance, ('Distance' , 'PrintLength', *injured)].mean(axis=None)

filesDf.loc[pathId, ('Proportion', 'ToeSpread' )] = framesDf.loc[inStance, ('Proportion', 'ToeSpread' )].mean(axis=None)

filesDf.loc[pathId, ('Proportion', 'IntSpread' )] = framesDf.loc[inStance, ('Proportion', 'IntSpread' )].mean(axis=None)

filesDf.loc[pathId, ('Proportion', 'PrintLength' )] = framesDf.loc[inStance, ('Proportion', 'PrintLength' )].mean(axis=None)

filesDf.loc[pathId, ('SFI' )] = framesDf.loc[inStance, ('SFI' )].mean(axis=None)

# Save frame data to file.

outputPath = path.parent / path.stem.replace('DLC', 'SFI')

if 'h5' in framesOutputTypes:

framesDf.to_hdf(str(outputPath.with_suffix('.h5')), key='df', mode='w')

if 'csv' in framesOutputTypes:

framesDf.to_csv(str(outputPath.with_suffix('.csv')), index=False)

# Save averages to a single file.

filesDf['Filename'] = [path.as_posix() for path in paths] if saveFullPath else [path.stem for path in paths]

outputPath = Path(projectFolder) / meansFilename

if 'h5' in meansOutputTypes:

filesDf.to_hdf(str(outputPath.with_suffix('.h5')), key='df', mode='w')

if 'csv' in meansOutputTypes:

filesDf.to_csv(str(outputPath.with_suffix('.csv')), index=False)

# Path to project folder.

projectFolder = r'/Users/NicolasL/Desktop/PNI37DigiGait_DLC/Baseline2/'

# Output filename file average: Will save into project folder with appropriate file extension.

meansFilename = r'MeanSFIBaseline2'

# Whether to include full path for each averaged file or just the name.

saveFullPath = False

# Output types: ('csv',) or ('h5',) or ('csv', 'h5')

framesOutputTypes = ('csv', )

meansOutputTypes = ('csv', )

# Minimum confidence for target body parts to calculate average.

confidence = 0.9

# Side of injured.

injurySide = 'Right'

process(projectFolder, meansFilename, saveFullPath, framesOutputTypes, meansOutputTypes, confidence, injurySide)
