# Supplementary material for: Predegenerating donor nerve for grafting using focused ultrasound neurotomy
Source: Sci Rep. 2025 May 4;15:15581. doi: 10.1038/s41598-025-00316-8 (PMC12050296; doi:10.1038/s41598-025-00316-8)
Supplement: Supplementary file 2 — Supplementary Material 2 [file 41598_2025_316_MOESM2_ESM.docx]

**Supplementary Figures**

**
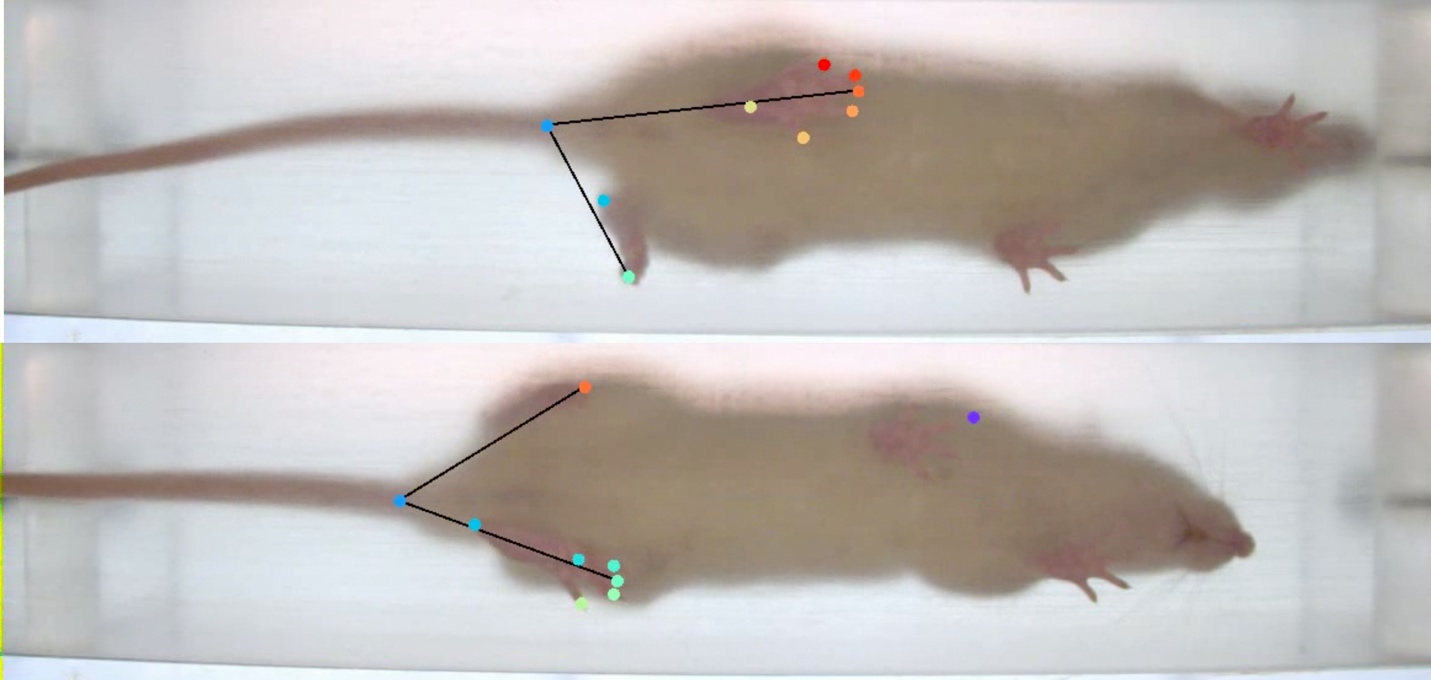
**

**Δ Supp Fig 1**

Representative footage captures of automated tracking of digits show precise identification of digits in contralateral foot (top) and ipsilateral foot (bottom) during walking.


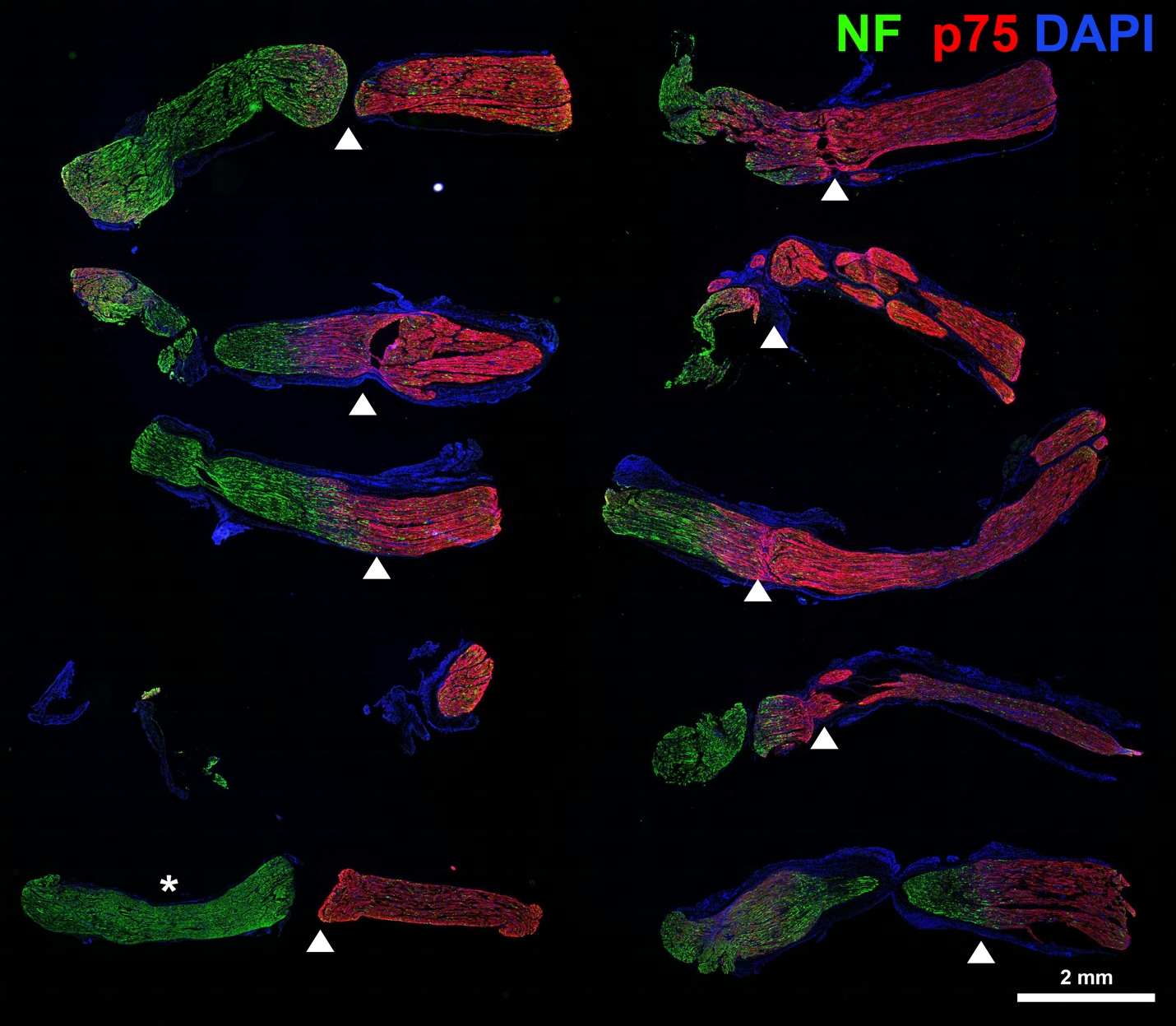


**Δ Supp Fig 2**

Immunostaining of the ten predegenerated nerves from donor Lewis rats for 6-week transplantation studies. The short segments include the focal lesions (arrowheads) and distal ends to right which show strong p75 expression, indicating complete axotomy at the HIFU focal point. A sample from contralateral normal nerve (asterisk) is shown to show absence of p75 staining, which is also evident in the left nerve segments proximal to the HIFU lesion in each case.


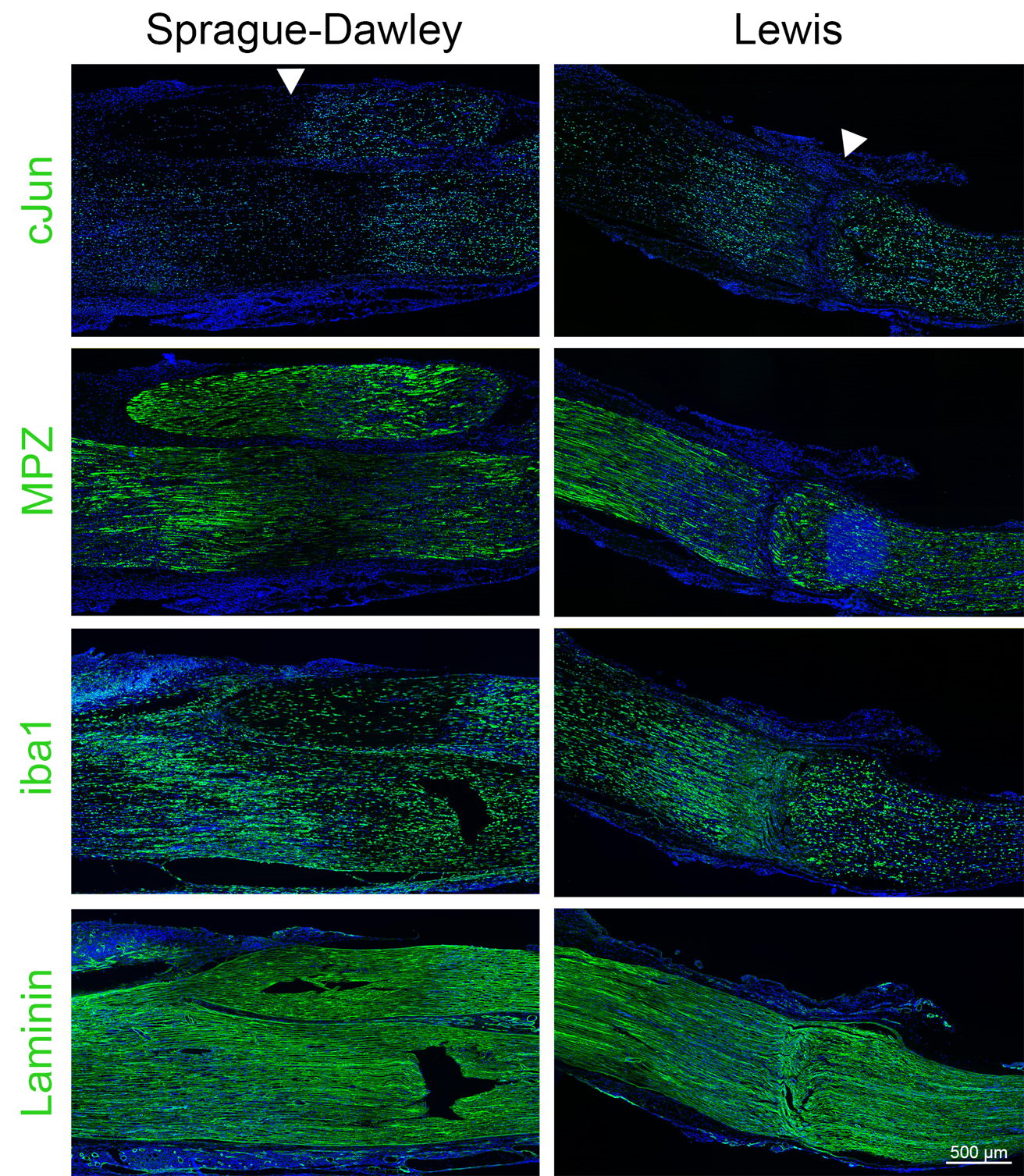


**Δ Supp Fig 3**

Immunostaining of nerves treated with high-intensity focused ultrasound in Sprague-Dawley and Lewis rats revealed similar patterns of cellular responses. The sonication site is indicated by an arrowhead. Both rat strains exhibited comparable Schwann cell activation, as demonstrated by the repair Schwann cell marker c-Jun, myelin debris at the distal end labeled with myelin protein zero (MPZ), macrophage infiltration indicated by Iba1 staining, and extracellular matrix highlighted by laminin expression.

**
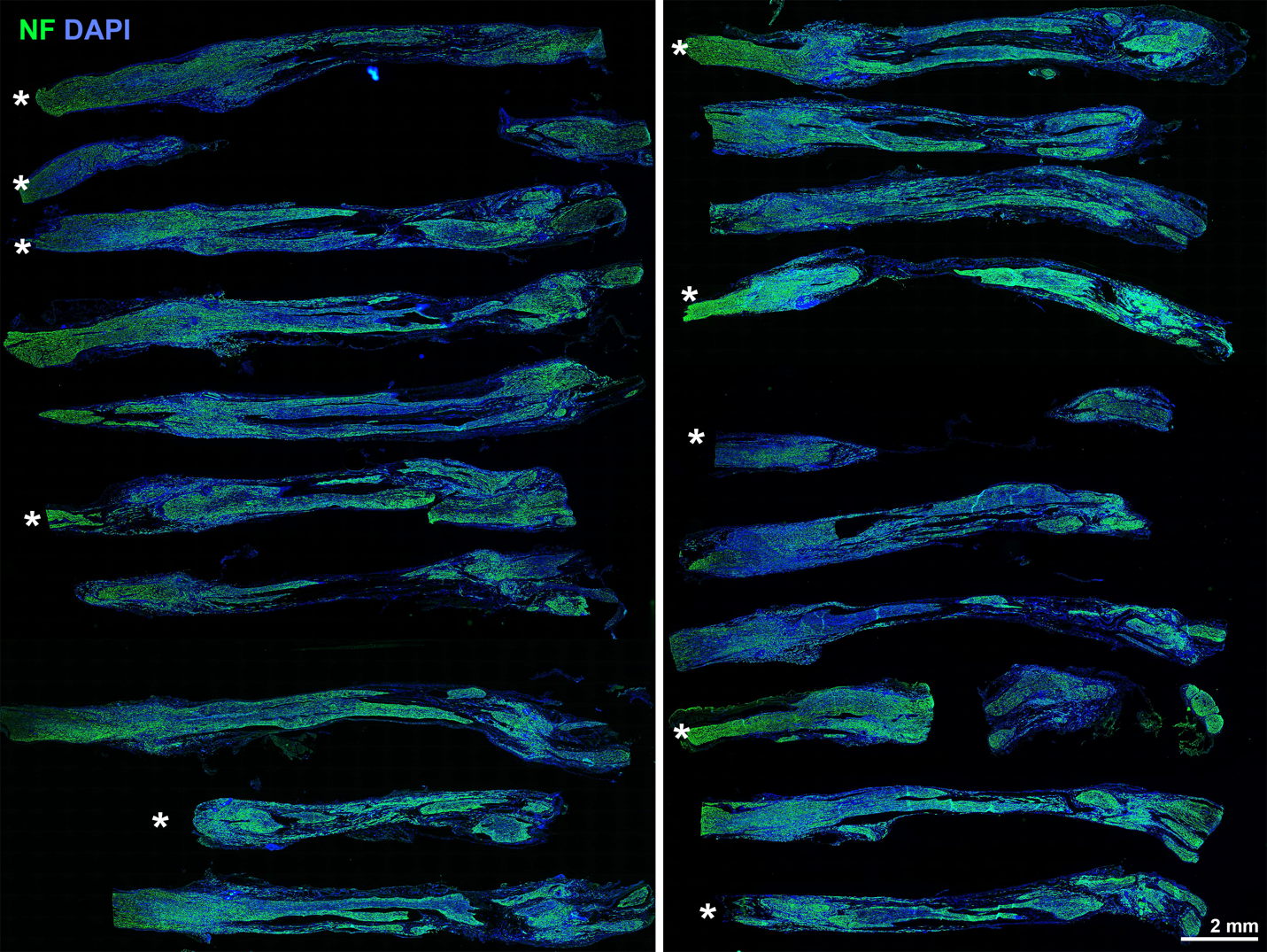
**

**Δ Supp Fig 4**

Immunostaining of all the grafts from the 20 animals for 6-week transplantation studies. The nerve grafts were labeled with neurofilament (NF, green) antibody which show smooth transition across neurorrhaphy sites. Nerve with predegenerated grafts were marked with asterisks.


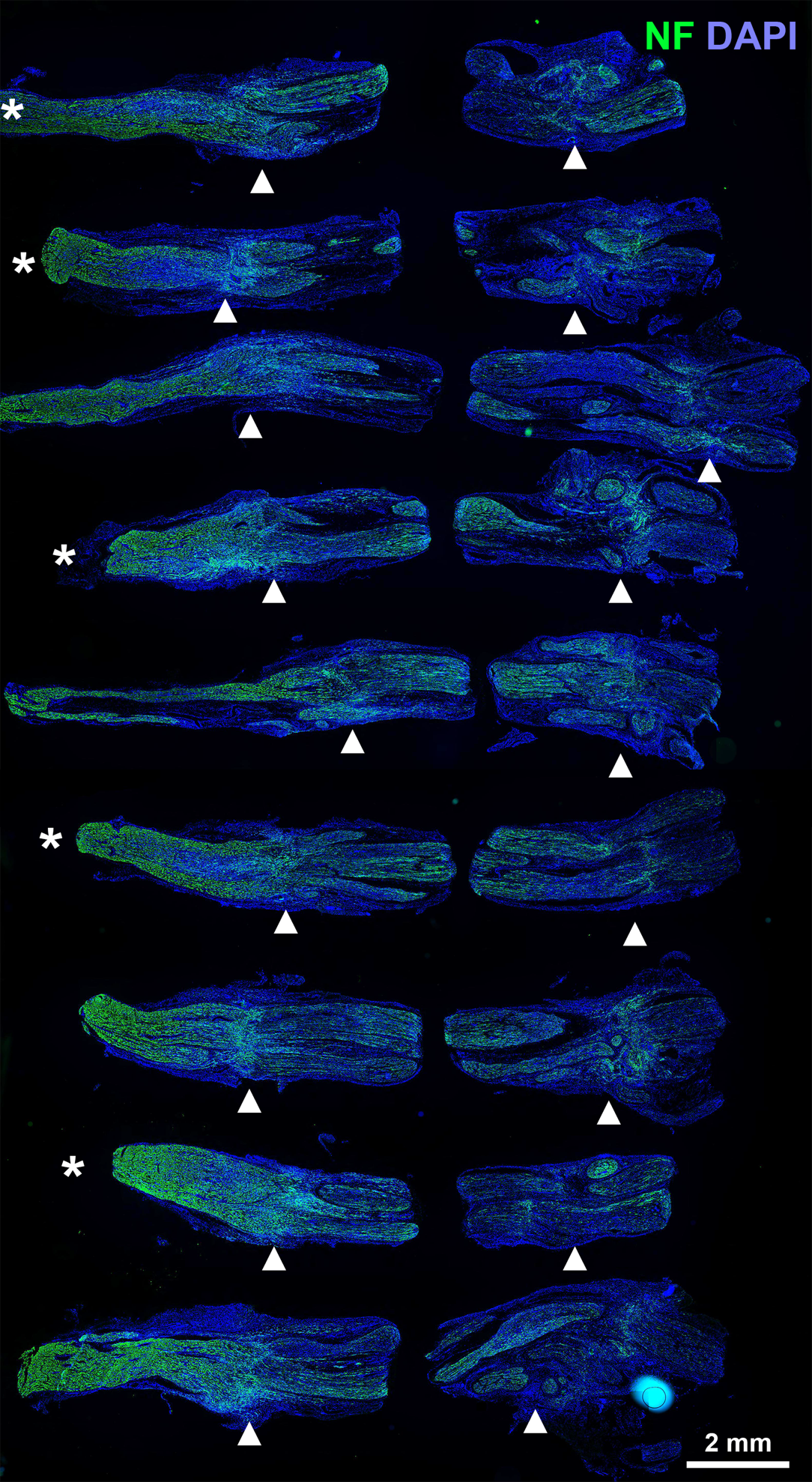


**Δ Supp Fig 5**

Immunostaining of all the grafts from the 9 animals for 2-week transplantation studies (one sample was missing for immunostaining). The nerve grafts were labeled with neurofilament (NF, green) antibody which show smooth transition across neurorrhaphy sites (arrowheads). Nerve with predegenerated grafts were marked with asterisks. Mid-grafts were harvested for semithin histomorphometry and therefore the interruptions in the middle.
